# Effects of Lipopolysaccharide-Binding Protein (LBP) Single Nucleotide Polymorphism (SNP) in Infections, Inflammatory Diseases, Metabolic Disorders and Cancers

**DOI:** 10.3389/fimmu.2021.681810

**Published:** 2021-07-06

**Authors:** Leilei Meng, Zichen Song, Anding Liu, Uta Dahmen, Xiao Yang, Haoshu Fang

**Affiliations:** ^1^ Department of Pathophysiology, School of Basic Medical Sciences, Anhui Medical University, Hefei, China; ^2^ Experimental Medicine Center, Tongji Hospital, Tongji Medical College, Huazhong University of Science and Technology, Wuhan, China; ^3^ Experimental Transplantation Surgery, Department of General, Visceral and Vascular Surgery, Friedrich-Schiller-University Jena, Jena, Germany

**Keywords:** inflammation, single nucleotide polymorphisms, lipopolysaccharide-binding protein, infections, inflammatory diseases, metabolic disorders, cancers

## Abstract

Inflammation, which is induced by the immune response, is recognized as the driving factor in many diseases, including infections and inflammatory diseases, metabolic disorders and cancers. Genetic variations in pivotal genes associated with the immune response, particularly single nucleotide polymorphisms (SNPs), may account for predisposition and clinical outcome of diseases. Lipopolysaccharide (LPS)-binding protein (LBP) functions as an enhancer of the host response to LPS, the main component of the outer membrane of gram-native bacteria. Given the crucial role of LBP in inflammation, we will review the impact of SNPs in the *LBP* gene on infections and inflammatory diseases, metabolic disorders and cancers.

## Highlights

- Inflammation, which is induced by the immune response, is recognized as the driving factor in many diseases, including infections and inflammatory diseases, metabolic disorders and cancers.
- Genetic variations in pivotal genes associated with the immune response, particularly single nucleotide polymorphisms (SNPs), account for predisposition and clinical outcome of inflammation-related diseases.
- Lipopolysaccharide (LPS)-binding protein (LBP) plays a crucial role in the innate immune response for the development of inflammatory and infectious-related diseases.
- This review aims to summarize the investigation and association of SNPs in the LBP gene with infections and inflammatory diseases, metabolic disorders and cancers.

## Introduction

In many diseases, including infections, metabolic disorders and cancers, inflammation is recognized as the driving factor (1–3). Functionally, inflammation is widely defined as a protective response against invading pathogens and endogenous stress signals (4), regulated by various pathways (such as TLR4/NF-kB pathway and IL-6/JAK/STAT3) and a multitude of genes (5–7). Interactions of cells from the innate and the adaptive immune system, together with the corresponding inflammatory mediators, contribute to various aspects of inflammation underlying many organ diseases (3, 8). Although the spectrum of inflammatory diseases is rather wide (ranging from systemic diseases such as sepsis, Type 1 diabetes and organ disease such as hepatitis) the cellular and molecular pathways involved in the inflammatory response remain similar (5).

Lipopolysaccharide, also called endotoxin, is a major component of the outer membrane of gram-negative (GN) bacteria (9). LPS causes endotoxemia when released into blood (10). Release of high levels of LPS into the systemic circulation (or release of lower levels in case of LPS-sensitization), induces a systemic inflammatory response syndrome, also called SIRS. The development of SIRS aggravates any other concomitant inflammatory condition, e.g. contributes to the development of septic shock in cases of sepsis (10).

The inflammatory response to LPS is mediated *via* LPS binding protein LBP. Lipopolysaccharide (LPS)-binding protein (LBP) is mainly produced in hepatocytes, functions as a secretory class I acute-phase protein (11–13) and plays a pivotal role in the innate immune response. Initially, LPS binds to LBP forming the LPS-LBP complex, which subsequently binds to CD14 and to the MD-4/MD-2 complex (14), ultimately results in the activation of signal transduction pathways and the production of cytokines and other pro-inflammatory mediators (15). Circulating LBP has a concentration-dependent immunologic function. Subnormal levels of LBP protein enhance phagocytosis and clearance of LPS from blood (16). In contrast, at high concentrations, LBP protein attenuates the release of pro-inflammatory cytokines (17). Additionally, LBP transfers LPS to very-low-density lipoproteins, low-density lipoproteins, high-density lipoproteins or chylomicrons, contributing to immune reaction against the infection (18–22). LBP also contributes to the immune response to gram-positive bacteria and fungal infection (23–25).

The physiological concentration of LBP in the serum of healthy human individuals is 5 to 10μg/mL (26). In contrast, during sepsis, up to seven-fold higher levels were observed within 24h (27). Increased LBP levels were also induced by a diet rich in fat and carbohydrates (28). Therefore, individuals suffering from obesity, diabetes, and related metabolic disorders also present with highly elevated LBP-levels (29–31), which was probably attributed to chronic low-grade inflammation induced by alterations of the intestinal flora together with an increased gut permeability to LPS (32, 33).

LBP is encoded by the *LBP* gene (34). The human *LBP* gene locates on chromosome 20 and shares an intron-exon pattern with other lipid-binding and transferring proteins on the same chromosome (34, 35). Genetic variations in *LBP* may contribute to susceptibility and clinical course of infectious diseases (36). On the basis of the crucial function of LPS in the innate immune response, many studies were performed to the genetic variation in the *LBP* gene in respect to the development of inflammatory diseases.

Single nucleotide polymorphisms (SNPs) are the most common type of genetic variation. The term refers to sequence alternatives (alleles) of a single DNA base with a frequency of more than 1% (37). SNPs influence the risk for the development and the outcome of a disease *via* various mechanisms but they are not considered to cause diseases (38). In coding regions, nonsynonymous SNPs could generate an amino-acid exchange and in turn affect the structure and biological function of the encoded protein, whereas synonymous SNPs do not lead to amino-acid exchange (39). Gu et al. reported that both synonymous and nonsynonymous SNP led to abnormal gene expression *via* altering the structure of local messenger RNA (38). In addition, mutations in the promoter region usually result in reduced mRNA levels (37). John C has demonstrated in his previous study that SNPs occurred in less conserved regions more frequently and the lowest occurrence rate was at the splice sites (40).

Evidence accumulated that genetic variations in pivotal genes associated with the immune response, particularly SNPs, may account for predisposition and clinical outcome of many diseases and potentially allow to predict prognosis and therapeutic effects (36, 41, 42).

In this review, we aim to present the current knowledge regarding the occurrence of SNP in the LBP gene and the impact of SNP in the *LBP* gene on the development and the course of selected infections, inflammatory diseases, metabolic disorders and cancers.

## Correlation Between SNP in LBP Gene and Infections, Inflammatory Diseases, Metabolic Disorders and Cancers

### LBP-SNPs in Infections

Evidence is accumulating that specific SNPs in the LBP gene are associated with a different course of many infections, including sepsis, infectious complications, and some organs infectious diseases (see in **Table 1**).

**Table 1:** Impact of SNPs in LBP gene on infectious diseases, sepsis and septic complications.

dpSNP ID	Disease	SNP Carrier	Main statement	Reference
-(Cys98→Gly)	sepsis	–	Gly98 allele may contribute to an increased predisposition to sepsis in male patients	(43)
rs2232582	septic complications after HCT	Heterozygous	C allele associated with a 2-fold higher risk for GN bacteremia after HCT.	(21)
	Infective endocarditis	Heterozygous	T allele may associate with high risk for infective endocarditis.	(44)
rs2232596	Infective endocarditis	Homozygous	G allele may associate with high risk for infective endocarditis.	(44)
a 4-SNP haplotype:	sepsis	Homozygous	The haplotype associated with increased risk for sepsis and higher mean levels of serum LBP.	(45)
rs1780616-C				
rs5741812-A				
rs2232571-T				
rs1780617-A				
rs2232571	septic complications after HCT	Heterozygous	C allele was associated with higher median basal serum LBP levels, a 3-fold increase in the risk of death prior to discharge, and a 5-fold increase in mortality risk.	(21)
	hematopoietic cell transplantation	Heterozygous	C allele was associated with higher median basal serum LBP levels.	(46)
rs2232613	hematopoietic cell transplantation	Heterozygous	Showed association with lower plasm LBP concentrations but higher efficiency in endotoxin extraction and transfer to MD-2.	(46)
	pneumonia and sepsis	Heterozygous	Carriage of T allele impaired bacterial ligands binding capacity and cytokines-induction function and exhibited a higher risk for mortality in the course of septic complications and pneumonia.	(47)
rs2232618	trauma	–	Generated mutant protein had an enhanced binding capacity with CD14. C allele contributed to higher incidence and development of sepsis.	(48)
	Sepsis	Homozygous	A combination with additional four SNPs was associated with sepsis in children.	(49)
	trauma	Heterozygous	C allele was associated with and higher incidence and development of sepsis.	(50)
	bacterial vaginosis	–	It was marginally significantly associated with Carriage of A. vaginae during early pregnancy.	(51)

Hubacek et al. reported that a gender-related polymorphism, a nonsynonymous SNP at the 292nd nucleotide of the *LBP* gene, was associated with an increased predisposition to sepsis in males (p<0.02) but not in female patients (43). Compared with controls, a significantly higher proportion of male individuals in the cohort of patients with sepsis carried at least one Gly98 allele (43). Interestingly, Barber and colleague determined this SNP (T292G) was not exist by pyrosequencing, which occurred due to adjacent nucleotide substitution (T→C) at 291 position. Therefore, the association was thought to be false-positive (52). In addition, Barber et al. reported T291C lacked association with septic complications and survival after trauma (52).

Rs2232582 (T291C) is a synonymous variant located in the coding region, which conferred a nucleotide substitution from T to C. Both variants encoded the amino acid at residue 97 (52). Chien and colleagues used a SNP haplotype tagging approach tagSNP in a retrospectively case-control population with 97 patients and 204 controls (21). They reported the C allele of rs2232582 (tagSNP 6878) was associated with GN bacteremia after hematopoietic cell transplantation (HCT) in multivariate analysis (p=0.001) (21). And in whites, the C allele was identified to be associated with a 2-fold higher risk for GN bacteremia (p=0.002) (21). Additionally, Vollmer et al. studied five LBP-SNPs in 78 infective endocarditis (IE) patients and 156 healthy blood donors, among which T allele of rs2232582 (291C>T) had a weak association with an increased susceptibility to infective endocarditis (p=0.019) (44). However, the significance didn’t remain after Bonferroni correction (291C>T pc=0.095) (44).

Rs2232596 is a synonymous variant and confers A→G nucleotide substitution at the 613rd position. Chien et al. reported rs2232596 (SNP 17002) was associated with GN bacteria in multivariate analysis in HCT (p=0.027) (21). Vollmer et al. determined the Gallele of rs2232596 (613A>G) was associated with an increased susceptibility to infective endocarditis(p=0.018) (44). Similar with rs2232582 in this research,no significance remained after Bonferroni correction (Pc=0.090) (44).

Rs2232571, which confers a C→T nucleotide substitution at -778 position in a CAAT box, locates in the promotor region of *LBP* gene (21) and regulates promoter efficiency (11). Chien et al. described that the SNP is in strong linkage disequilibrium with rs2232582 aforementioned (21). In a prospective cohort of 234 cases, patients of HCT carrying rs2232571 (SNP1683) C allele significantly displayed a 3-fold increase in the risk of death prior to discharge (p=0.001) (21). In the development of GN bacteremia, mortality risk of patients significantly increased five-fold when they carried C allele of rs2232571 (p=0.013) (21). Moreover, rs2232571 C allele carriers exhibited higher median basal serum LBP levels (CC, 17.39μg/mL; TC 10.4μg/mL; TT 8.07μg/mL, p=0.004) (21). In addition, Guinan et al. confirmed previous linkage between rs2232571and plasm LBP levels in HCT (46). Flores and colleagues explored nine polymorphisms of LBP gene in the 5’-flanking region which is the transcription start site from position -1978 to -763. They observed no association between these SNPs along with the incidence of severe sepsis (45). Notably, a common 4-SNP haplotype consisting of allelic combination rs1780616-C/rs5741812-A/rs2232571-T/rs1780617-A was significantly associated with the predisposition to severe sepsis (p=0.016) (45). In comparison with non-carriers, homozygous carriers showed an increased risk of sepsis (p=0.016) and higher mean serum LBP concentrations at inclusion (130.1μg/mL for homozygous carriers and 101.6μg/mL for non-carriers, respectively) and at the seventh day (98.9μg/mL for homozygous carriers and 58.7μg/mL for non-carriers, respectively. p=0.046) (45).

Rs2232613 is a nonsynonymous mutation, which leads to proline changed to leucine at residue 333 (47). Eckert and colleagues described the structure of murine LBP and revealed the presence of rs2232613 affected a “phenylalanine core” in the C-terminal of the LBP and resulted in a dysfunction protein (47). The mutant protein reduced bacterial ligands binding capacity and cytokines-induction function *in vitro* (47). It was consistent with the results *in vivo* experiments that the variant T allele carriers displayed lower cytokine levels during experimental LPS stimulation. For example, at 1hr after injection of LPS, the production of TNF-α of heterozygous mutation carriers is significantly lower (p=0.013) (47). Carriers produced lower cytokine concentrations when suffering from ventilator-associated pneumonia (6.2 ± 1.76pg/mL for carriers and 18.4 ± 4.9 pg/mL for WT individuals, respectively. P=0.014) (47). A retrospective clinical analysis of 424 ICU surgical patients indicated that T allele carriers exhibited higher risk for mortality (CT/TT vs. CC, 11.5% vs. 5.8%, p=0.1) (47). Statistically significant difference was also seen in mortality in patients with infectious complications (CT/TT vs. CC, 21.2% vs. 8.9%, p=0.02 for hospital-acquired pneumonia; 33.3% vs. 8.2%,p=0.03 for hospital-acquired pneumonia caused by Gram-negative bacteria) (47). Studies demonstrated that *LBP* deficiency protected mice from LPS stimulation but exhibited higher lethality in bacterial stimulation, which might account for that carriers of rs2232613 exhibited higher risk of severe clinical infection (47). Guinan et al. also confirmed T allele carriers of rs2232613 displayed lower plasm LBP concentrations but higher efficiency in endotoxin extraction and transfer to MD-2 in HCT patients (46).

Rs2232618, is a functional variant conferring an amino acid substitution from phenylalanine to leucine at residue 436 caused by a T→C nucleotide substitution in the C-terminal domain of the LBP protein (48). According to the research conducted by Hubacek and colleagues, no positive association between rs2232618 with the occurrence of sepsis was described (43). However, Zeng et al. assessed the entire LBP gene in major trauma cohorts in Chinese population *via* using haplotype tagging SNPs (htSNPs) and detected that among the nine htSNPs studied in LBP, only rs2232618 associated with traumatic sepsis (48). According to 557 trauma patients in Chongqing cohort, C allele carriers showed a higher of sepsis and MODS (TT vs. TC+CC, p<0.0001 for sepsis morbidity rate and p=0.011 for MOD scores, respectively), when compared with T allele carriers (48). TNFα production by peripheral leukocytes of C allele carriers *in vitro* revealed a significantly increased response to LPS (TT vs. TC, p=0.01). The clinical relevance of genotypic distribution of rs2232618 was further confirmed in Zhejiang cohort with 230 patients (TT vs. TC, p=0.012 for sepsis morbidity rate and p=0.046 for MOD scores, respectively) (48). In vitro experiments, the binding capacity with LPS (p<0.001) and TNFα production (p<0.001) were significantly enhanced with the presence of the variant Leu436 LBP protein. Furthermore, Leu436 LBP protein has stronger binding interactions with CD14 (p=0.043) (48). Lu and their groups confirmed the correlation between genotypic distributions and traumatic sepsis in an enlarged co-located population sample size with 1296 patients in Chongqing (TT vs. TC+CC, p=0.002 for sepsis morbidity rate and p=1.8E-6 for MOD scores, respectively) and 445 patients in Zhejiang (TT vs. TC+CC, p=0.002 for sepsis morbidity rate and p=0.005 for MOD scores, respectively) (50). Combined analysis of two cohorts also showed relevance between rs2232618 and traumatic sepsis (TT vs. TC+CC, p=4.5×10^-4^ for sepsis morbidity rate and p=1.4E-9 for MOD scores, respectively) (50). In addition, Jabandziev et al. reported rs2232618 in combination with additional four SNPs (*BPI* rs574350, *TLR* rs4986790, *HSP70* rs2227956, *IL-6* rs1800795) could be regarded as a predictor of clinical outcome of sepsis in children (49). The authors defined wild-type homozygotes as A, heterozygotes and mutant homozygotes as B. Among analyses of *BPI*, *TLR* and *LBP*, *LBP* A + *BPI* A + *TLR* A was associated with a high risk of sepsis (p=0.005), *LBP* B + *BPI* A + *TLR* A was a high-risk combination for sepsis with severe condition (p=0.034) (49). On the contrary, *LBP* A + *BPI* B + *TLR* A was a low-risk combination for both sepsis and severe condition (p<0.001 and p=0.003, respectively) (49). In terms of combinations of *LBP*, *IL-6* and *TLR*, *LBP* A + *IL-6* B +*TLR* A is a low-risk combination of severe sepsis (p=0.027) (49). Combinations of *LBP* A + *IL-6* A +*TLR* B and *LBP* B + *IL-6* A +*TLR* A were regarded to be a high risk for sepsis (p<0.006 and p=0.012, respectively), but the proportion of patients was low (4.2% and 4.8%, respectively) (49). Analysis of five genes showed a combination of wild-type homozygotes was associated with high risk of sepsis. Moreover, *BPI* A + *LBP* A + *TLR* A +HSP70 A +IL-6 B was also a high-risk combination for sepsis (p=0.016) (49). On the contrary, *BPI* B + *LBP* A + *TLR* A +HSP70 A +IL-6 B and *BPI* B + *LBP* A + *TLR* B +HSP70 A +IL-6 B were low-risk combinations for both sepsis (p=0.006 and p=0.041, respectively) and sepsis with severe condition (p=0.001 and p=0.026) (49). In addition, Verstraelen et al. observed rs2232618 was marginally significantly associated with carriage of *Atopobium vaginae* in bacterial vaginosis during early pregnancy (p=0.056) (51).

### LBP-SNPs in Atopy and Asthma

Atopy is viewed as a predisposition to the immune response to diverse antigens*/*allergens, inducing CD4+ Th2 differentiation and overproduction of immunoglobulin E (IgE) (53). Asthma is a manifestation of atopy, which is a chronic inflammatory airway disease caused by combined influence of genetic and environmental factors (54). Asymptomatic atopic individuals, healthy individuals or atopic individuals without current manifestation of disease exhibited increased serum LBP levels after inhaling LPS. These findings suggest that atopy and asthma related phenotype might be associated with LBP genetic variants (55). Two studies related to asthma reported six SNPs of LBP. In the coding region, rs2232596 is a synonymous mutation and rs2232580 is a missense mutation that confers an amino acid substitution from proline to leucine at residue 9. Moreover, rs6025083 and rs12624843 are located in the intron, rs5741812 is located in the 5’-UTR and rs745144 is located in the 3’-UTR. Reijmerink and colleagues and colleagues demonstrated gene-environment interactions of atopy in children (56). Rs2232596, day-care and one sibling were selected as the best three-factor model (p=0.007) (56). Day-care protected children from developing high total IgE at 1-2 years old who were heterozygotes for rs2232596, but not in children with more than one sibling (56). rs745144, in combination with sibling and CD14 (rs2915863) were selected as the there-factor predictors for total IgE at 6-8 years old (56). Besides, rs6025083, along with TLR1 (rs5743594) and TLR5 (rs851186) were selected as three-factor predictors of gene-gene interaction of specific IgE to indoor allergens 6–8 years old (56). Daley et al. reported rs2232580 and rs5741812 associated with atopy in children with a family history of allergies (p=0.0141 and p=0.423, respectively) (57). In the healthy study cohort, rs12624843 were associated with airway hyperresponsiveness (AHR) (p=0.0443) (57). Additionally, SNP-virus interaction analyses showed rs5741812 was significantly associated with AHR and picornavirus (p=0.048) (57) (see in **Table 2**).

**Table 2:** Effect of SNP in LBP-gene atopy, metabolic diseases and cancer.

Disease	dpSNP ID	Effect	Reference
atopy	rs2232596	It was one of the predictors of total IgE at 1-2 years old in the three-factor model.	(56)
	rs745144	It was one of the predictors of total IgE at 6-8 years in the three-factor model.	
	rs6025083	It was one of the predictors of specific IgE to indoor allergens at 6–8 years old in the three-factor model.	
asthma	rs2232580	Two SNPs associated with atopy in children with a family history of allergies.	(57)
	rs5741812		
	rs12624843	It was associated with airway hyperresponsiveness (AHR).	
type 2 diabetes	rs1739654	Four SNPs were significantly correlated with susceptibility to type 2 diabetes. Rs2232592 indicated a risk for diabetes with the history of obesity and/or earlier age of onset.	(58)
	rs2232578		
	rs2232592 rs1780627		
HIV-1/HAART-associated lipodystrophy syndrome (HALS)	rs2232582	It had significant association with HALS, T allele having greater prevalence of HALS.	(59)
myocardial infarction	G292→T	It had no significant association with a risk of myocardial infarction.	(60)
	rs2232618		
	(Pro436Leu)		
cerebral infarction	rs2232582	Two SNPs had significant association with Carotid intima-media thickness (CIMT) in Chinese, but both were not associated with risk of atherosclerotic cerebral infarction.	(61)
	(291T>C)		
	rs2232618		
	(1341T>C)		
colorectal carcinoma	rs2232596	G allele had a significant association with an increased CRC risk.	(62)
colorectal neoplasia	rs1780617	G allele was significantly associated with the risk of CRC and adenoma.	(63)
gastric cancer	rs2232578	It was associated with the risk of GC in combination with H. pylori infection.	(64)
glioma	rs2232580	It to glioma risk and susceptibility.	(65)

### LBP-SNPs in Metabolic Disorders

**I**ncreasing evidence suggested gut microbiota and low-grade inflammation are associated with chronic diseases, such as metabolic disorders (66), cardiovascular disease (67) and hypertension (68). Given the involvement of LBP in inflammation (67), some SNPs have been investigated in related diseases (see in **Table 2**).

Rs1739654 is a synonymous mutation located in the coding region, rs2232578 is located in the 5’-UTR, rs1780627 and rs2232592 are located in intron region. Takeuchi et al. identified this four SNPs of LBP gene on chromosome 20q significantly correlated with susceptibility to type 2 diabetes p=0.001 for rs1739654, p=0.003 for rs1780627, p=0.011 for rs2232592 and p=0.0004 for rs2232578, respectively (58). Further stratified analysis for rs2232592 indicated diabetic patients with the history of obesity and/or earlier age of onset have prominent genetic risk (58).

As previously mentioned, rs2232582 is a synonymous variant and confers T→C nucleotide substitution. Vilade's et al. reported rs2232582 was associated with HIV-1/HAART-associated lipodystrophy syndrome (HALS). Carriage of T allele was significantly associated the higher incidence of HALS whereas carriage of C allele seemed to protect individuals against HALS (p=0.01) (59). Besides, Zhan et al. reported 291T>C (rs2232582) was associated the Carotid intima-media thickness (CIMT) (p<0.05), but not cerebral infarction in Chinese population (61). CIMT is considered to be a risk of cardiovascular and cerebrovascular disease and a surrogate marker to assess AS (69), patients carrying TC genotype exhibited higher mean CIMT compared with TT genotype (61).

As the leading cause of myocardial infarction and stroke (70), atherosclerosis (AS) is a complex disease and involves a series of pathophysiological processes such as inflammation and lipid metabolism (71). Hubacek et al. demonstrated that the frequency of LBP 1306T>C (rs2232618) C allele (Leu436) homozygotes is about threefold in patients with myocardial infarction than in controls (60). But the frequency of this genotype in controls is very low and did not reach statistical significance (2.3% in patients and 0.7% in controls, respectively) (60). In addition, Zhan et al. reported 1306T>C (rs2232618), similar to rs2232582 in the study, was associated with CIMT (p<0.05) (61). Individuals with TC genotype exhibited higher mean CIMT compared with TT genotype (61).

### LBP-SNPs in Cancer

Inflammation is closely related to the pathogenesis of cancer (72, 73) and genetic variations of inflammatory regulators were demonstrated to associate with the increased risk or adverse consequence of several tumors (74–79).

Intestinal microbiota dysbiosis leads to more release of LPS and thereby to a higher stimulation of LBP and subsequent production of inflammatory cytokines (75). Chronic low-grade inflammation contributes to carcinogenesis and the development of colorectal carcinoma (CRC) (80). Chen et al. demonstrated that mutant G allele of rs2232596 was significantly associated with the CRC risk in patients compared with healthy controls (GA vs. AA, p=0.003; GG vs. AA, p=0.016; GA+GG vs. AA, p=0.001, respectively). When combined with CD14 rs4914, the risk of CRC increased 3.4 times (p=0.000) (62). Additionally, both smokers carrying GA genotype and drinkers carrying GA or GG genotype had an increased risk for CRC (p=0.005 for smokers and p=0.001 for drinkers, respectively), which revealed the interaction between genes with tobacco and alcohol (62). According to another study conducted by Chang et al, rs1780617 G allele was significantly associated with the risk of CRC and adenoma, a precursor to CRC (p=0.046) (63).

*Helicobacter pylori (HP)* infection accounts for the progression of gastric cancer (GC) (76). Castaño-Rodríguez et al. demonstrated that rs2232578, which is located in 5’-UTR, induced an increased risk for GC in combination with *HP* infection in Chinese population (p=0.0292 for AA and p=0.017 for G carriers) (64).

LEE et al. conducted a Genome-wide pathway analysis and identified six SNPs, five genes and nine pathways in glioma. Rs2232580 contributed to the risk of glioma *via* affecting the interaction of LBP to LPS and immune responses (0.002≤p ≤ 0.013) (65).

The overview of analysis of LBP SNPs which exhibit association in inflammation-related diseases is shown in **Table 3** classified with SNP ID. In addition, SNPs that were investigated but did not show correlation are summarized in **Table 4**.

**Table 3:** Summary of Lipopolysaccharide Binding Protein (LBP) SNPs correlated with corresponding disease.

dpSNP ID	Allele	Phenotype	Author	Disease/Topic	Year	Population	Patients/controls or total size	Reference
rs2232613	998C/T	*P333L*	Eckert	pneumonia and septic complications	2013	Germany	424	(47)
			Guinan	hematopoietic cell transplantation	2014	American	249	(46)
rs2232618	1341T/C	*F436L*	Verstraelen	bacterial vaginosis	2009	Caucasian	144	(51)
			Zeng	trauma	2012	Chinese	787	(48)
			Zhan	cerebral infarction	2012	Chinese	366/200	(61)
			Jabandziev	sepsis	2014	Czechs	598/529	(49)
			Lu	trauma	2018	Chinese	1741	(50)
–	T292→G	*C98G*	Hubacek	sepsis	2001	–	204/250	(43)
rs2232580	C/A,T	*P9L*	LEE	glioma	2015	European	1856/4955	(65)
			Daley	Asthma	2012	Canadian and Australian	5565	(57)
rs2232582	291T/C	synonymous variant	Chien	septic complications after HCT	2008	Multiethnic	350/865	(21)
			Vollmer	infective endocarditis	2009	–	78/156	(44)
			Zhan	cerebral infarction	2012	Chinese	366/200	(61)
			Vilades	HIV-1/HAART-associated lipodystrophy syndrome	2014	Multiethnic	240/318	(59)
rs2232596	613A/G	synonymous variant	Vollmer	infective endocarditis	2009	–	78/156	(44)
			Reijmerink	atopy	2011	Dutch	3062	(56)
			Chen	colorectal carcinoma	2011	Chinese (Han)	479/486	(62)
rs1739654	G/A	synonymous variant	Takeuchi	type 2 diabetes	2007	Japanese	675/474	(58)
rs2232571	C/T	promotor region	Chien	septic complications after HCT	2008	Multiethnic	350/865	(21)
			Flores	sepsis	2009	European	175/357	(45)
			Guinan	hematopoietic cell transplantation	2014	American	249	(46)
rs2232578	A/G	promotor region	Takeuchi	type 2 diabetes	2007	Japanese	675/474	(58)
			Castaño-Rodríguez	gastric cancer	2014	Chinese	87/223	(64)
rs1780616	C/T	5’-region	Flores	sepsis	2009	European	175/357	(45)
rs5741812	A/T	promoter region	Flores	sepsis	2009	European	175/357	(45)
			Daley	Asthma	2012	Canadian and Australian	5565	(57)
rs1780617	A/G	5’-region	Flores	sepsis	2009	European	175/357	(45)
			Chang	colorectal neoplasia	2013	American	1139/696	(63)
rs745144	C/G,T	3’-region	Reijmerink	atopy	2011	Dutch	3062	(56)
rs2232592	G/A	intron	Takeuchi	type 2 diabetes	2007	Japanese	675/474	(58)
rs6025083	C/T	intron	Reijmerink	atopy	2011	Dutch	3062	(56)
rs12624843	G/A	intron	Daley	Asthma	2012	Canadian and Australian	5565	(57)

**Table 4:** Summary of Lipopolysaccharide Binding Protein (LBP) SNPs not associated with corresponding disease.

dpSNP ID	Allele	Phenotype	Author	Disease/Topic	Year	Population	Patients/controls or total size	Reference
rs2232613	998C/T	*P333L*	Kozlowski	baseline CRP levels	2006	Caucasian	717	(81)
			Verstraelen	bacterial vaginosis	2009	Caucasian	144	(51)
			Mare-Bredemeijer	liver transplantation	2013	Caucasian	671	(82)
			Haerynck	cystic fibrosis	2013	Belgian	96/79	(83)
rs2232618	1341T/C	*F436L*	Hubacek	sepsis	2001	American	204/250	(43)
			Torok	inflammatory bowel disease	2004	Caucasians	102+98/145	(84)
			Vollmer	infective endocarditis	2009	German	78/156	(44)
			Chen	colorectal carcinoma	2011	Chinese (Han)	479/486	(62)
			Poikonen	*Chlamydia pneumoniae* infection	2009	Finnish	258	(85)
			Mare-Bredemeijer	liver transplantation	2013	Caucasians	671	(82)
			Gonzalez-Quintela	LBP concentration	2013	Caucasians	420	(86)
			Haerynck	cystic fibrosis	2013	Belgian	96/79	(83)
			Bloudickova	End-Stage Renal Disease	2014	Caucasians	1014/2559	(87)
			Vilades	HIV-1/HAART-associated lipodystrophy syndrome	2014	Multiethnic	240/318	(59)
rs2232580	C/A,T	*P9L*	Zeng	trauma	2012	Chinese	787	(48)
rs2232607	A/G	*D283G*	Verstraelen	bacterial vaginosis	2009	Caucasian	144	(51)
			Mare-Bredemeijer	liver transplantation	2013	Caucasian	671	(82)
rs5744204	G/A	*V166M*	Verstraelen	bacterial vaginosis	2009	Caucasian	144	(51)
			Mare-Bredemeijer	liver transplantation	2013	Caucasian	671	(82)
	T292G	*C98G*	Hubacek	myocardial infarction	2002	Czech	313/302	(60)
rs2232596	613A/G	synonymous variant	Chien	septic complications after HCT	2008	Multiethnic	350/865	(21)
			Flores	sepsis	2009	European	175/357	(45)
			Castaño-Rodríguez	gastric cancer	2014	Chinese	87/223	(64)
			Vilades	HIV-1/HAART-associated lipodystrophy syndrome	2014	Multiethnic	240/318	(59)
rs2232582	291T/C	Synonymous variant	Barber	trauma	2003	Multiethnic	151/133	(52)
			Torok	inflammatory bowel disease	2004	Caucasian	102+98/145	(84)
			Bloudickova	End-Stage Renal Disease	2014	Caucasian	1014/2559	(87)
rs1739654	G/A	synonymous variant	Chen	colorectal carcinoma	2011	Chinese (Han)	479/486	(62)
rs2232571	C/T	Promotor region	Zeng	trauma	2012	Chinese	787	(48)
rs2232578	A/G	Promotor region	Kozlowski	baseline CRP levels	2006	Caucasian	717	(81)
			Chien	septic complications after HCT	2008	Multiethnic	350/865	(21)
			Flores	sepsis	2009	European	175/357	(45)
			Verstraelen	bacterial vaginosis	2009	Caucasian	144	(51)
			Mare-Bredemeijer	liver transplantation	2013	Caucasian	671	(82)
			Haerynck	cystic fibrosis	2013	Belgian	96/79	(83)
rs1780616	C/T	5’-region	Kozlowski	baseline CRP levels	2006	Caucasian	717	(81)
			Chien	septic complications after HCT	2008	Multiethnic	350/865	(21)
			Vollmer	infective endocarditis	2009	German	78/156	(44)
			Zeng	trauma	2012	Chinese	787	(48)
rs5741812	A/T	5’-region	Kozlowski	baseline CRP levels	2006	Caucasian	717	(81)
rs2425361	A/C	5’-region	Zeng	trauma	2012	Chinese	787	(48)
rs2425364	G/T	5’-region	Zeng	trauma	2012	Chinese	787	(48)
rs2232573	T/C	5’-region	Flores	sepsis	2009	European	175/357	(45)
rs5741811	T/A,C	5’-region	Kozlowski	baseline CRP levels	2006	Caucasian	717	(81)
rs745144	C/G,T	3’-region	Kozlowski	baseline CRP levels	2006	Caucasian	717	(81)
rs1780623	T/C	intron	Zeng	trauma	2012	Chinese	787	(48)
rs11536972	T/C	intron	Zeng	trauma	2012	Chinese	787	(48)
rs6127841	C/T	intro	Zeng	trauma	2012	Chinese	787	(48)
rs2298266	C/T	intron	Takeuchi	type 2 diabetes	2007	Japanese	675/474	(58)
rs2298267	G/A	intron	Takeuchi	type 2 diabetes	2007	Japanese	675/474	(58)
rs3819023	A/G	intron	Takeuchi	type 2 diabetes	2007	Japanese	675/474	(58)
rs1739640	C/T	intron	Takeuchi	type 2 diabetes	2007	Japanese	675/474	(58)
rs1780627	C/T	intron	Verstraelen	bacterial vaginosis	2009	Caucasian	144	(51)
			Haerynck	cystic fibrosis	2013	Belgian	96/79	(83)
			Mare-Bredemeijer	liver transplantation	2013	Caucasian	671	(82)
			Takeuchi	type 2 diabetes	2007	Japanese	675/474	(58)
rs6099236	C/G,T	intron	Kozlowski	baseline CRP levels	2006	Caucasian	717	(81)
rs6025083	C/T	intron	Kozlowski	baseline CRP levels	2006	Caucasian	717	(81)
rs11536945	G/A,C	intron	Kozlowski	baseline CRP levels	2006	Caucasian	717	(81)
rs11536949	G/T	intron	Kozlowski	baseline CRP levels	2006	Caucasian	717	(81)
			Flores	sepsis	2009	European	175/357	(45)
rs5741817	T/C	intron	Kozlowski	baseline CRP levels	2006	Caucasian	717	(81)
rs2232602	G/A	intron	Kozlowski	baseline CRP levels	2006	Caucasian	717	(81)
rs11536988	T/A	intron	Kozlowski	baseline CRP levels	2006	Caucasian	717	(81)
			Flores	sepsis	2009	European	175/357	(45)

## Results of Meta-analysis

**Figure 1:**
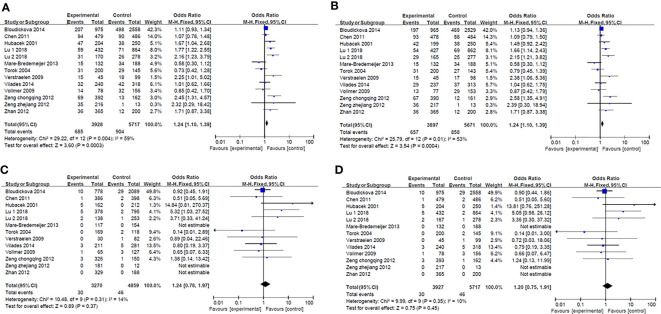
Forest plot in rs2232618. **(A)** Dominant model (TT+TC *vs* CC). **(B)** heterozygote model (TC *vs* CC). **(C)** homozygotes models (TT *vs* CC). **(D)** recessive models (TT *vs* TC + CC).

**Figure 2:**
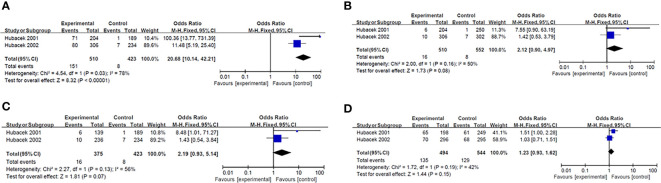
Forest plot in synonymous 292. **(A)** Dominant model (TT+TG *vs* GG). **(B)** heterozygote model (TG *vs* GG). **(C)** homozygotes models (TT *vs* GG). **(D)** recessive models (TT *vs* TG +GG).

**Figure 3:**
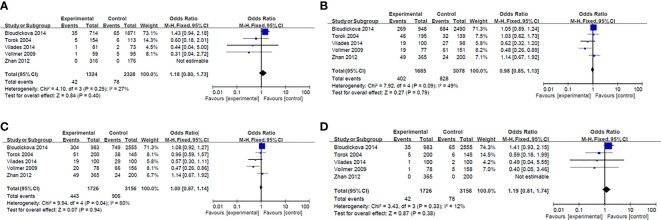
Forest plot in rs2232582. **(A)** Dominant model (TT+TC *vs* CC). **(B)** heterozygote model (TC *vs* CC). **(C)** homozygotes models (TT *vs* CC). **(D)** recessive models (TT *vs* TC + CC).

Results of smeta -analysis of susceptibility associated with rs2232618, synonymous 292 and rs2232592 in diseases are performed in **Figures 1**–**3**, respectively.

## Conclusion

Characterization of genetic predisposition underlying diseases is the prerequisite for identifying patients at risk and for developing specific therapeutic strategies. In this review we give evidence for the relationship between SNPs in the *LBP* gene and certain inflammatory diseases.

Our finding might help to identify individuals at risk for inflammatory complications by genotyping and thereby contribute to developing more widely applicable diagnostic strategies. Furthermore, our finding might represent one step towards designing therapeutic strategies based on genetic heterogeneity using e.g. KO technologies.

However, more clinical studies including much higher patient numbers are needed to reach statistical significance confirming the reported trends as well as for confirming the postulated relationships in independent patient cohorts.

In addition, identifying high-risk individuals based on genotype and adjusting therapeutic strategies timely in the clinic could improve the prognosis of patients.

However, our findings are limited in three aspects: reproducibility of results and size of patient cohorts. So far, only two of the reported association between LBP-polymorphism and disease association were replicated by an independent study. Thus, additional studies are needed to verify the association between a certain SNP and susceptibility for a given disease.

Some of the reported positive but also negative associations were obtained in small patient cohorts (see in **Table 3** and **Table 4**), which calls for confirmatory studies investigating larger groups of patients. Doing so will lead to sound results which can be used for developing novel therapeutic approaches, e.g., using pharmacological KO strategies.

## Author Contributions

Data collection: LM, ZS and HF. Manuscript preparation: LM, AL and HF. Critical revision: LM, UD, XY and HF. All authors contributed to the article and approved the submitted version.

## Funding

This work was supported by National Science Foundation of China (No. 81971875) and the University Natural Science Foundation of Anhui Province (No. KJ2019A0220)

## Conflict of Interest

The authors declare that the research was conducted in the absence of any commercial or financial relationships that could be construed as a potential conflict of interest.

## Abbreviations

LBP, lipopolysaccharide binding protein; LPS, lipopolysaccharide; SNP, Single nucleotide polymorphism; GN, Gram-negative; HCT, hematopoietic cell transplantation; htSNPs, haplotype tagging SNPs; AHR, airway hyperresponsiveness; HALS, HIV-1/HAART-associated lipodystrophy syndrome; AS, atherosclerosis; CIMT, Carotid intima-media thickness; CRC, colorectal carcinoma; HP, *Helicobacter pylori;* GC, gastric cancer.
